# The Role of Phoneme in Mandarin Chinese Production: Evidence from ERPs

**DOI:** 10.1371/journal.pone.0106486

**Published:** 2014-09-05

**Authors:** Mengxia Yu, Ce Mo, Lei Mo

**Affiliations:** Center for the Study of Applied Psychology, South China Normal University, Guangzhou, China; University of Akron, United States of America

## Abstract

Established linguistic theoretical frameworks propose that alphabetic language speakers use phonemes as phonological encoding units during speech production whereas Mandarin Chinese speakers use syllables. This framework was challenged by recent neural evidence of facilitation induced by overlapping initial phonemes, raising the possibility that phonemes also contribute to the phonological encoding process in Chinese. However, there is no evidence of non-initial phoneme involvement in Chinese phonological encoding among representative Chinese speakers, rendering the functional role of phonemes in spoken Chinese controversial. Here, we addressed this issue by systematically investigating the word-initial and non-initial phoneme repetition effect on the electrophysiological signal using a picture-naming priming task in which native Chinese speakers produced disyllabic word pairs. We found that overlapping phonemes in both the initial and non-initial position evoked more positive ERPs in the 180- to 300-ms interval, indicating position-invariant repetition facilitation effect during phonological encoding. Our findings thus revealed the fundamental role of phonemes as independent phonological encoding units in Mandarin Chinese.

## Introduction

Language production involves a series of consecutive stages: Initially, the intended message is formed at the conceptual preparation stage, followed by speech planning during which lexical items are selected and combined according to the grammar of the language, and finally articulated. According to the language production model by Levelt, Roelofs and Meyer [1], a critical phase in this process is the phonological encoding during which the sound forms of the lexical items in the intended message are formulated by inserting and sequencing phonemes or clusters of phonemes (i.e. phonological encoding units) into syllabic metrical frames. The idea that this process takes place in units of phonemes has been supported by many studies of Indo-European languages. Using implicit priming techniques, researchers found that alphabetic speakers were faster at naming target words with the same initial phoneme as the prime in comparison to those sharing no phonological commonality with the prime, e.g., hut-*h*oop and hut-*p*ool in Dutch [2]–[4], even when they were not aware of the prime words [5]–[7]. Moreover, studies employing a colored picture-naming task in which subjects named the visual objects using color-object phrases have revealed performance improvement either when the initial (e.g., /b/ in '*b*lack *b*ox') or the non-initial phoneme (e.g., /a/ in 'bl*a*ck p*a*n') was identical between the color adjective and the object noun [8], [9]. Together, these findings showed that oral production of alphabetic lexicons was consistently facilitated by overlapping phonemes irrespective of their specific in-word position, suggesting that phonemes serve as the basic functional units in phonological encoding in alphabetic system.

In contrast, there is no consensus concerning the phonological encoding units in logographic languages. On one hand, it has been proposed that syllable and mora, rather than phoneme, serve as the prominent processing unit in spoken Chinese and Japanese respectively, as a result of the natural differences in phonological features across languages [10]. This was supported by earlier behavioral findings of robust syllabic repetition priming effects in the absence of priming effects induced by overlapping single phonemes during Mandarin word production [10]–[13]. Moreover, segmental priming effects were not observable in Japanese speech production either in implicit priming [14] or masked priming tasks [15]. On the other hand, it was argued that phonemes are the primary functional units in Chinese phonological encoding. In support of this idea, a recent electrophysiological study found that when overseas Chinese students performed a colored picture-naming task (see above), color-object phrases with common onsets (phoneme repetition condition) elicited more positive ERPs in the 200- to 300-ms time window than those with different onsets (non-repetition condition), indicating a robust phonemic repetition priming effect. The researchers further proposed that the reason for the absence of phonemic priming effects on naming responses might be the enhanced internal self-monitoring interference effect triggered by increased susceptibility to speech errors, as the same phoneme was retrieved repeatedly within a portion of utterance, e.g., /h/ in “*h*uang2 *h*e2 zi (yellow box)” [16], [17]. Moreover, the sub-syllabic priming effect was also observed when highly proficient Mandarin Chinese-English bilinguals were engaged in a masked priming-naming task in Chinese [18]. Hence, the functional units of phonological encoding in Mandarin Chinese still remain controversial and elusive.

**Figure 1:**
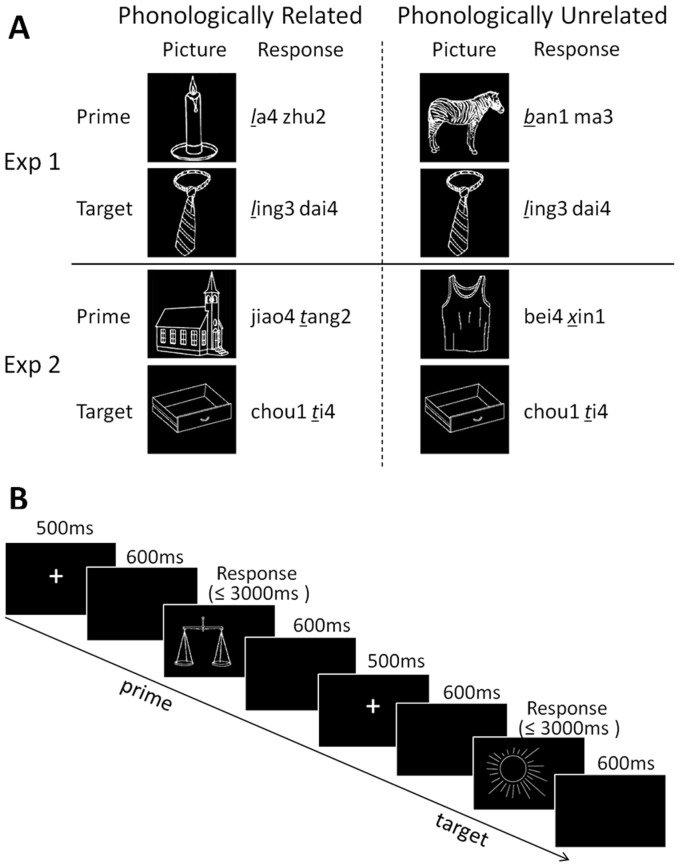
Examples of experimental stimuli (A) and Experimental paradigm (B) . (A) For the phonologically related condition, the first (Experiment1) or the second (Experiment2) consonant in the Mandarin Chinese disyllabic names of prime picture and target picture were identical. For the phonologically unrelated condition, the corresponding manipulated phoneme was different. Numbers represent tone. (B) Each experimental trial was comprised of a naming procedure for the prime picture and then a naming procedure for the target picture.

Despite the potential involvement of phonemes in Chinese speech production, two critical issues remain unaddressed. To begin, the reported phonemic priming effect might arguably result from the idiosyncratic sensitivity to phonemes brought by English proficiency in bilingual Chinese participants, which implicitly affected the phonological encoding process in Mandarin Chinese [18], [19]. Moreover, the observed phonemic repetition facilitation might be another instance of the so-called “initialness effect”, which refers to the special role of word-initial segments in speech production, e.g. initial phonemes are more frequently involved in speech errors such as segmental exchange, e.g., *l*eft *h*emisphere - *h*eft *l*emisphere [20], [21], since there is no evidence of priming effects induced by phonological overlap in a single non-initial phoneme during Chinese phonological encoding. Here, we addressed these two issues by systematically investigating the initial and non-initial phoneme priming effect in native Chinese speakers in two ERP experiments. To enable the effective manipulation of phonemic repetition, we adopted a picture-naming priming task which required the participants to pronounce a set of words corresponding to the visually presented pictures in each trial [9], [10], [22], [30], [31]. Furthermore, to minimize the confounds introduced by complex interaction between facilitation and inhibition when employing traditional implicit priming paradigm with multi-item stimuli sets, we limited the size of each set to two disyllabic words which were referred to as prime and target respectively (Fig. 1B). The experimental design involved two conditions: 1) the phonologically related condition in which the initial consonant (Experiment 1) or the second consonant (Experiment 2) of the paired disyllabic picture names were identical; 2) the phonologically unrelated condition in which the corresponding manipulated consonants of the prime and the target words were different (Fig. 1A). If phonemic priming is caused by enhanced sensitivity to phonemes in proficient Chinese-English bilinguals, no facilitation effect would be found in either experiment because representative native Chinese speakers were not characterized by fluency in alphabetic languages. If phonemic priming is related to the “initialness effect”, one would expect the phonemic priming effect on presentation of identical initial phonemes during phonological encoding stage, which is reported to occur from 180 ms after picture onset [16], [23]. Critically, if phonemes serve as fundamental functional units in phonological encoding of Mandarin Chinese, one would predict repetition priming effects for overlapping phonemes in both the word-initial and non-initial positions during Chinese speech production.

## Results

### Behavioral results

Trials in which participants stuttered, failed to respond or made incorrect response to either the prime picture or the target picture (about 0.45% and 1.26% in experiment 1 and 2 respectively) were excluded. In addition, trials with naming latencies exceeding 1400 ms or shorter than 400 ms (Experiment 1: 2.40%; Experiment 2: 3.64%) were also excluded from subsequent analyses. Mean naming latencies of target words in the trials surviving the above exclusion criteria were analyzed using repeated measures ANOVA with phonological relatedness as the within-subject factor, with separate analyses conducted across participants (F1) and items (F2). Overall naming errors were infrequent (< 2%) and were approximately equal between the two conditions. Hence, no further analyses were conducted on the error rates.

In the first experiment, we found no significant difference in naming latencies between the word pairs with overlapping initial phoneme and phonologically unrelated word pairs (Mean latencies: phonologically related condition: 807.08 ms, phonologically unrelated condition: 808.81 ms; *F1*(1,25)  =  0.14, *p*  =  0.714; *F2*(1,17)  =  0.05, *p*  =  0.830; *min F′*(1,29)  =  0.04, *p* > 0.1). Similarly, repetition of non-initial phonemes did not facilitate the naming of the target pictures (Mean latencies: phonologically related condition: 799.68 ms, phonologically unrelated condition: 792.55 ms; *F1*(1,25)  =  1.81, *p*  =  0.190; *F2*(1,17)  =  2.36, *p*  =  0.143, *min F′* (1,42)  =  1.02, *p* > 0.1). As can be seen, our behavioral results were in agreement with the absence of behavioral priming effects reported in previous studies of Chinese speech production.

### ERP results

ERP data analyses were conducted on electrophysiological waveforms evoked by target pictures naming in all effective trials after averaging three proximal electrodes to isolate six regions of interest (ROIs) along the sagittal and the coronal cerebral axes according to previous studies [16], i.e., left-anterior (F5, F7, FC5), mid-anterior (Fz, FCz, Cz), right-anterior (F6, F8, FC6), left-posterior (P5, P7, CP5), mid-posterior (CPz, Pz, POz), and right-posterior (P6, P8, CP6). We analyzed ERP data for the two experimental conditions in two time windows, 180–300 ms (the phonemic repetition facilitation effect) and 350–450 ms (the self-monitoring inhibitory effect), in order to directly test for the presence of the two effects for word-initial and non-initial overlapping phonemes. A 2 × 6 repeated measures ANOVA with phonological relatedness and ROI location as within-subject factors was conducted on the data pertaining to each of the two time windows respectively. Degrees of freedom were adjusted using Greenhouse-Geisser correction to account for data non-sphericity.

#### Repetition priming effects induced by overlapping phonemes

**Figure 2:**
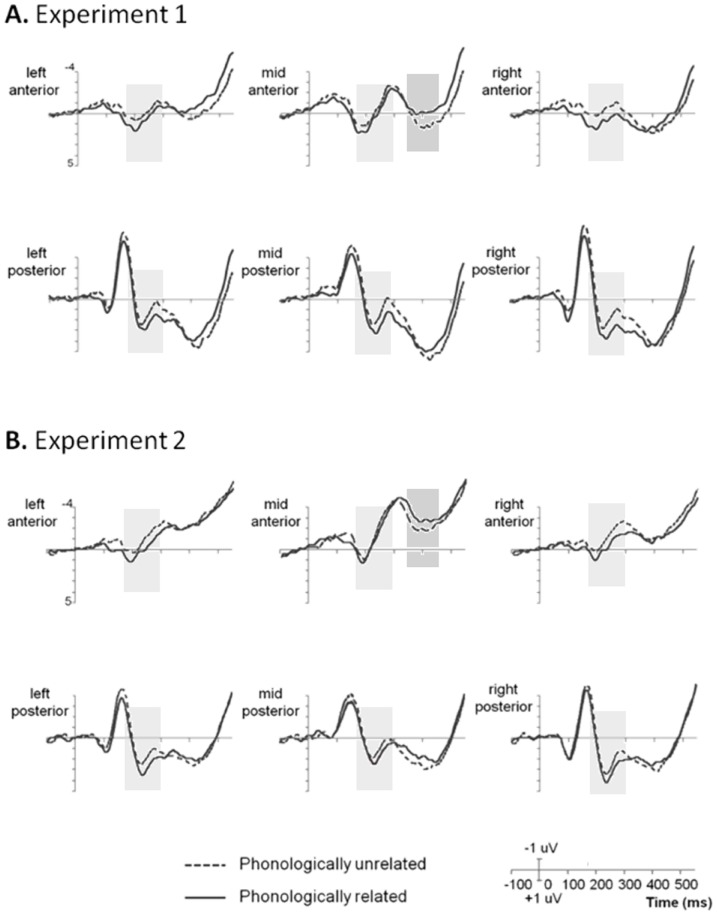
Grand average ERPs at six ROIs in Experiment 1 (A) and Experiment 2 (B). The origin of the time-axis (0 ms) is placed at the onset of the target pictures. ERPs elicited in the phonologically related condition (solid line) were significantly more positive than the unrelated condition (dotted line) in the 180- to 300-ms interval after picture onset across six ROIs (light grey shading), indicating that overlap of either initial (Experiment 1) or non-initial phonemes (Experiment 2) facilitates early speech planning in Mandarin Chinese production. An inversion of the phonological repetition effect was found in the 350- to 450-ms time window in the mid-anterior region (dark grey shading), suggesting later self-monitoring inhibition.

Grand average ERP waveforms pertaining to the phonologically related and unrelated conditions in the two experiments are plotted for each ROI in Fig. 2A and Fig. 2B respectively. Results of Experiment 1 showed that word pairs with overlapping initial phonemes elicited more positive ERPs than did the phonologically unrelated pairs (*F*(1,25)  =  6.30, *p*  =  0.019) in the 180- to 300-ms time window, indicating a repetition priming effect induced by initial phoneme overlap. Moreover, neither the main effect of ROI (*F*(5,125)  =  1.09, *p* > 0.1) nor the interaction between phonological relatedness and ROI (*F*(5,125)  =  2.23, *p* > 0.1) was found significant. These findings gave support to the results reported in earlier studies and further demonstrated the functional contribution of word-initial phoneme to Mandarin disyllable production.

Critically, in Experiment 2 we identified significant signal increase for the non-initial phoneme repetition condition across six ROIs from 180 ms to 300 ms after targets onset (*F*(1,25)  =  5.45, *p*  =  0.028). Similar to the first experiment, the repetition priming effect did not interact with the ROI location (*F*(5,125)  =  1.34, *p* > 0.1). In addition, there was a significant main effect of ROI location (*F*(5,125)  =  6.51, *p*  =  0.007). Taken collectively, these results expanded the previous findings by revealing a comparable repetition facilitation effect induced by the same phonemes in the non-initial position as was found for the initial position.

#### Inhibition effects induced by internal self-monitoring

In Experiment 1, we found significant interaction between initial phoneme relatedness and ROI location from 350 ms to 450 ms (*F*(5,125)  =  3.34, *p*  =  0.034), suggesting an inhibitory effect on neural activity resulting from inherent speech monitoring processes, which is consistent with previous findings [16]. Moreover, there was a significant main effect of the ROI, (*F*(5,125)  =  10.51, *p* < 0.001), but no statistical difference between the phonologically related and unrelated condition (*F*(1,25)  =  0.82, *p* > 0.1). To more specifically characterize the spatial distribution of the inhibition effect, post-hoc paired *t* tests were conducted to compare the 350- to 450-ms component for the two conditions in each ROI. We found more negative ERPs for the phonologically related condition than the unrelated condition, which was significant in mid-anterior region (*t*(25)  =  2.19, *p*  =  0.038) and marginally significant in left-anterior region (*t*(25)  =  1.90, *p*  =  0.069), while there was no significant difference between the two conditions in other areas (*p*s > 0.1). To further investigate this effect, additional analyses were conducted on data recorded from a larger set of electrodes across mid-anterior region (F1, Fz, F2, FC1, FCz, FC2, Cz, C2) using a 2 (phonologically relatedness) × 8 (electrodes) repeated measures ANOVA. We found a similar main effect of phonological relatedness (*F*(1,25)  =  4.84, *p*  =  0.037) which did not interact with electrode location (*F*(7,175)  =  0.89, *p* > 0.1).

In Experiment 2, we also identified a similar interaction between non-initial phoneme relatedness and ROI that was marginally significant between 350 ms and 450 ms (*F*(5,125)  =  2.78, *p*  =  0.057). The main effect of ROI was significant (F(5,125)  =  19.30, *p* < 0.001) while no main effect of relatedness was found (*F*(1,25)  =  0.92, *p* > 0.1). Since the interaction effect was very close to statistical significance, and there was a clear trend observable in mid-anterior region similar to Experiment 1, we conducted post-hoc paired t-test at each ROI to examine this effect in further detail. We found a significant inhibition effect in mid-anterior region as reflected by more negative ERPs for phoneme repetition condition (*t*(25)  =  2.21, *p*  =  0.037) and no statistical difference in other regions (*p*s > 0.1). Identical results were obtained in the additional analyses on a larger set of mid-anterior electrodes (F1, Fz, F2, FC1, FCz, FC2, Cz, C2). Similar to our previously reported results in Experiment 1, we found a significant main effect of relatedness (*F*(1,25)  =  4.28, *p*  =  0.049) and no Relatedness × Electrodes interaction (*F*(7,175)  =  1.17, *p* > 0.1).

## Discussion

We identified functional involvement of sound-sized segments in Chinese speech production by comparing the electrophysiological activity changes induced by phonologically related and unrelated picture pairs. We found more positive ERPs induced by overlapping initial and non-initial phonemes from 180 ms to 300 ms after stimulus onset across six ROIs, reflecting robust repetition priming effects during phonological encoding. Moreover, we also found more negative ERPs in phonemic repetition condition in the mid-anterior regions in the 350- to 450-ms interval, suggesting an inhibition effect triggered by stronger speech self-monitoring. Our work extends the previous findings of phonemic contribution to Chinese speech production and provides systematic neural evidence in support of the essential role of the phoneme as the fundamental phonological encoding unit in Mandarin Chinese.

Consistent with previous findings [16], we identified stronger positive electrophysiological signal evoked by phonologically related words from 180 ms to 300 ms, a critical period for phonological encoding that has been demonstrated in a number of overt naming studies [23]–[25]. Previous work found that the EEG signal was modulated at a latency of 180 ms after target word onset by the lexical frequency effect that is commonly thought to occur at the phonological encoding stage [23]. ERP responses elicited by a cumulative semantic interference effect (CSIE) that possibly spills over to phonological encoding process were modulated from 200 ms after picture onset and lasted for 180 ms [24]. Furthermore, enhanced positive ERP waveforms evoked by phonologically related words in a similar 250- to 400-ms interval after target onset were observed in German speakers performing a delayed picture-naming task [25]. In another picture-word interference study, researchers found positive ERPs in a similar time interval when distractor words shared the first two or three phonemes with the picture names [26]. Therefore we conclude that the positive ERP component we observed in the 180- to 300-ms time window reflected phoneme repetition priming that was related to the phonological encoding process.

Also, our results showed that phoneme repetition elicited stronger negative ERPs from 350ms to 450ms in the mid-anterior regions, which is in agreement with the finding of neural inhibition caused by internal self-monitoring to prevent speech errors [16]. Similar enhanced negative ERPs that occur subsequently after phonological encoding have been shown to be relevant for speech self-monitoring in several earlier studies. In a study using a go/no-go task to explore the metrical encoding and syllabification processes during language production, a greater negative ERP component associated with self-monitoring was identified in no-go trials which recruit more mental resources for cognitive regulation and control [27]. Further evidence comes from an MEG study, which also identified that the facilitatory effect of semantic relatedness during lexical selection was reversed in the subsequent self-monitoring time frame [28]. Based on these findings, the inversion effect we observed in the 350–450 ms interval is potentially related to the inherent self-monitoring mechanism in speech production. Additionally, our findings may provide a potential interpretation for null effect in previous behavioral implicit priming studies [10], [11]. As Qu et al [17] predicted, the self-monitoring mechanism which is also required in this task might cancel out subtle segment-based facilitation arising during phonological encoding. The naming process might initially be facilitated by repeated retrieval of the same phonemes but was subsequently hindered by increased cognitive workload in response to increased speech error probability, which leads to the absence of observable behavioral improvement.

However, an additional question arises why there was no similar behavioral phonemic priming effect in Chinese as in alphabetic languages. e.g., naming picture sets “day, dye, dough,dew” is faster than naming “day, pea, rye, sow” in English native speakers [10]. We propose that the cause for this cross-linguistic divergence might be the different cognitive weighting of sound-sized segments in logographic Chinese and other alphabetic languages. It has been proposed that syllables are the primary selectable phonological units below the word level in Chinese production, while phonemes are retrieved as independent functional units via mediation of syllables [10], [16], [18], [29]. Mandarin syllables are much fewer in quantity and relatively simpler in phonological structure when compared to syllables in alphabetic languages such as English and Dutch. For fluent Chinese speakers, it is therefore functionally feasible to access the entire syllable, while for alphabetic language speakers it is much more efficient to represent thousands of lexical items with dozens of phonemes. Furthermore, syllabic boundaries are clear and well-defined in Chinese with no occurrences of resyllabification, making each syllable a relatively independent unit for storage that can be manipulated during phonological encoding, while in alphabetic languages comparable functional manipulability and independency is located on the phonemic level [11]. Finally, alphabetic lexical sound forms are characterized by robust grapheme-phoneme connection, while the phonology of logographic Chinese is defined at the monosyllabic level, with no sub-character components corresponding to any segmental sound units. Taken collectively, given the critical involvement of phonemes in phonological processing in Indo-European languages, we concluded that phoneme-based facilitation during phonological encoding is strong enough to overwhelm the naming response deceleration caused by internal self-monitoring. In spoken Mandarin Chinese, on the other hand, phonemic facilitation is relatively weaker and more susceptible to self-monitoring inhibition, rendering it difficult to be captured by behavioral measurements.

Finally, it is not surprising that the paired-picture naming task adopted in our study is somewhat different from the colored picture naming task in the previous ERP study by Qu et al [16], but nevertheless had similar behavioral results. In contrast, several behavioral studies have found that segmental overlap between trials tends to induce more inhibition than that within trials [9], [30], [31]. A possible account is that instead of monosyllabic words used in these studies, the current study used 36 disyllabic words with fewer repetitions, which may enhance the priming effect to some extent, since preparation benefits tend to be larger with less familiar and more complex items (see [4], [32]). Although our findings cannot directly validate these potential processing mechanisms, it is important to note that, combined with [16], our ERP results revealed reliable functional involvement of sound-sized segments in Chinese speech production independent of experimental paradigms.

In conclusion, our electrophysiological findings revealed position-invariant phonemic repetition priming effect unrelated to acquired phonemic sensitivity when native Chinese speakers were orally producing Mandarin disyllable words in a picture-naming priming paradigm, suggesting that phonemes universally serve as phonological encoding units in alphabetic languages and non-alphabetic Mandarin Chinese. Future work is needed to further examine the neural mechanisms underlying the effective integration of the individual units into a unified phonological representation.

## Methods

### Ethics statement

The current study was approved by the Academic committee of the School of Psychology at South China Normal University. All participants gave written informed consent before participating in the experiments.

### Participants

Two groups of 26 healthy undergraduate students of South China Normal University with normal or corrected-to-normal vision participated in Experiment 1 (16 females) and Experiment 2 (14 females) respectively. All participants were native Chinese speakers in non-English majors. Mispronunciation is common even for native Mandarin speakers, which might result in the absence of priming effects despite the presence of overlapping phonemes due to inaccurate encoding of the stimuli sound forms, e.g. “*l*a4 zhu2 (candle)” might be pronounced incorrectly as “*n*a4 zhu2”, hence no priming effect would occur if paired with “*l*ing3 dai4 (tie)”. To minimize the rate of mispronunciation, all participants were required to have achieved a degree of Class 2 Level B or above in National Mandarin Proficiency Test to ensure pronunciation accuracy.

### Stimuli

Thirty-six black-on-white line drawings for each experiment were chosen from a standardized picture database [33]. All the pictures were common objects with high naming consistency in Mandarin Chinese. The names of the selected pictures were all disyllables involving 18 different consonants (i.e., two pictures each) in the first (Experiment 1) or second (Experiment 2) syllable with no phonemic overlap between the two syllables in a word (e.g., word like “sha1 fa1 (sofa)” was excluded because of a common vowel). Different pictures were used in the two experiments to manipulate different consonants. Stimuli sets corresponding to the two experimental conditions were formed by pairing the selected pictures. For the phonologically related condition, the initial consonant (Experiment 1) or the second consonant of disyllable (Experiment 2) were held the same for the prime and target words, e.g., “*l*a4 zhu2 (candle)” - “*l*ing3 dai4 (tie)” and “jiao4 *t*ang2 (church)” - “chou1 *t*i4 (drawer)”. In the phonologically unrelated condition, the onset consonants were different in the paired words, e.g., “*b*an1 ma3 (zebra)” - “*l*ing3 dai4 (tie)” and “bei4 *x*in1 (waistcoat)” - “chou1 *t*i4 (drawer)” (Fig. 1A). The tone of the manipulated syllables in each pair was different and there was no orthographic similarity or semantic relatedness between the two pictures' names (See Appendix S1 and Appendix S2 for a complete list of stimuli). To eliminate other possible confounds related to stimuli ordering and pairing, we expanded the stimulus set by exchanging the prime and the target in the original 18 pairs such that each picture acted as both prime and target once, yielding 36 stimuli pairs pertaining to each of the two experimental conditions.

### Experimental procedures

Instructions and stimuli were presented using E-Prime 2.0 software. Both experiments were comprised of a learning phase and a testing phase with identical procedures. In the learning phase, participants were asked to view each of the 36 selected line drawings for 3000 ms twice and memorize the corresponding name of each object simultaneously presented below. Before testing, all participants were given an additional practice session in which they named all 36 stimuli individually presented in a sequential order. The naming procedure started with a 500 ms fixation, followed by a blank screen of 600 ms. Stimuli were presented subsequently after the offset of the blank screen and disappeared at the start of naming response or the end of maximal stimulus duration of 3000 ms. Consecutive naming procedures were separated by a fixed-length inter-trial-interval of 600 ms. Naming responses were collected with a microphone connected to the computer with a PST Serial Response Box. During the testing phase, participants were asked to produce a learned disyllable word to name the object as accurately and quickly as possible after the picture was displayed. Each experimental trial was comprised of a naming procedure for the prime picture and then a naming procedure for the target picture (Fig. 1B). The order of experimental conditions was counterbalanced across participants and the presentation of stimuli pairs was pseudo-randomized to minimize the possible phonological, semantic or orthographic relatedness between two consecutive trials. Each pair was presented twice, which thus resulted in a total of 144 trials (36 pairs × 2 conditions × 2 repetitions) for each participant. During the recording process, participants were asked to refrain from moving or coughing to reduce EEG artifacts. The entire experimental session lasted for approximately 30 minutes with two short breaks.

### ERP recordings

EEG activity was recorded continuously with scalp impedance constrained to be 5 kΩ or below throughout the recording session using Neuroscan SynAmps2 amplifier and 64 electrodes fitted on a Quick Cap based on the international 10–20 system. An electrode was placed on the participant's nose as the reference channel. Vertical EOG (VEOG) was recorded in 2 electrodes placed above and below the left eye respectively, while horizontal EOG (HEOG) was recorded by two electrodes placed at the bilateral outer canthi of eyes. EEG Recordings were sampled at a rate of 1,000 Hz and filtered off-line using a 40-Hz low-pass (zero-phase) and segmented into 650-ms epochs comprised of a 100-ms pre-stimulus baseline and a 550-ms post-stimulus interval, which was chosen to minimize speech contamination. Segments containing artifacts exceeding ± 100 µV were rejected before averaging. In each condition, mean amplitude ERPs for target pictures were computed.

## Supporting Information

Appendix S1 **Stimuli used in Experiment 1.**
(DOC)

Appendix S2 **Stimuli used in Experiment 2.**
(DOC)
