# Supplementary material for: The Role of Phoneme in Mandarin Chinese Production: Evidence from ERPs
Source: PLoS One. 2014 Sep 5;9(9):e106486. doi: 10.1371/journal.pone.0106486 (PMC4156350; doi:10.1371/journal.pone.0106486)
Supplement: Appendix S1 — Stimuli used in Experiment 1. (DOC) [file pone.0106486.s001.doc]

**Appendix S1: Stimuli used in Experiment 1.**Numbers represent tones and neutral tone is not marked.

| Phonologically related prime-target pairs | | Phonologically unrelated prime-target pairs | |
| --- | --- | --- | --- |
| 天平 (tian1 ping2, “balance”) | 太阳 (tai4 yang2, “sun”) | 灯泡 (deng1 pao4, “light bulb”) | 太阳 (tai4 yang2, “sun”) |
| 窗户 ( chuang1hu4, “window”) | 衬衫 (chen4 shan1, “shirt”) | 青蛙 (qing1 wa1, “frog”) | 衬衫 (chen4 shan1, “shirt”) |
| 蜡烛 (la4 zhu2, “ candle”) | 领带 (ling3 dai4, “tie”) | 斑马 (ban1 ma3, “zebra”) | 领带 (ling3 dai4, “tie”) |
| 青蛙 (qing1 wa1, “frog”) | 裙子 (qun2 zi, “skirt”) | 天平 (tian1 ping2, “balance”) | 裙子 (qun2 zi, “skirt”) |
| 树叶 (shu4 ye4, “leaf”) | 手枪 (shou3 qiang1, “gun”) | 蜡烛 (la4 zhu2, “ candle”) | 手枪 (shou3 qiang1, “gun”) |
| 酒杯 (jiu3 bei1, “wineglass”) | 箭头 (jian4 tou2, “arrow”) | 公鸡 (gong1 ji1, “rooster”) | 箭头 (jian4 tou2, “arrow”) |
| 灯泡 (deng1 pao4, “light bulb”) | 地球 (di4 qiu2, “globe”) | 窗户 ( chuang1hu4, “window”) | 地球 (di4 qiu2, “globe”) |
| 斑马 (ban1 ma3, “zebra”) | 背心 (bei4 xin1, “waistcoat”) | 树叶 (shu4 ye4, “leaf”) | 背心 (bei4 xin1, “waistcoat”) |
| 公鸡 (gong1 ji1, “rooster”) | 拐杖 (guai3 zhang4, “cane”) | 飞机 (fei1 ji1, “airplane”) | 拐杖 (guai3 zhang4, “cane”) |
| 钟表 (zhong1 biao3, “clock”) | 栅栏 (zha4 lan, “fence”) | 松鼠 (song1 shu3, “squirrel”) | 栅栏 (zha4 lan, “fence”) |
| 开关 (kai1 guan1, “light switch”) | 孔雀 (kong3 que4, “peacock”) | 南瓜 (nan2 gua1, “pumpkin”) | 孔雀 (kong3 que4, “peacock”) |
| 松鼠 (song1 shu3, “squirrel”) | 算盘 (suan4 pan2, “abacus”) | 蘑菇 (mo2 gu1, “mushroom”) | 算盘 (suan4 pan2, “abacus”) |
| 飞机 (fei1 ji1, “airplane”) | 纺车 (fang3 che1, “spinning wheel”) | 吸管 (xi1 guan3, “sucker”) | 纺车 (fang3 che1, “spinning wheel”) |
| 花生 (hua1 sheng1, “peanuts”) | 蝴蝶 (hu2 die2, “butterfly”) | 酒杯 (jiu3 bei1, “wineglass”) | 蝴蝶 (hu2 die2, “butterfly”) |
| 南瓜 (nan2 gua1, “pumpkin”) | 纽扣 (niu3 kou4, “button”) | 喷泉 (pen1 quan2, “fountain”) | 纽扣 (niu3 kou4, “button”) |
| 蘑菇 (mo2 gu1, “mushroom”) | 蜜蜂 (mi4 feng1, “bee”) | 开关 (kai1 guan1, “light switch”) | 蜜蜂 (mi4 feng1, “bee”) |
| 喷泉 (pen1 quan2, “fountain”) | 苹果 (ping2 guo3, “apple”) | 花生 (hua1 sheng1, “peanuts”) | 苹果 (ping2 guo3, “apple”) |
| 吸管 (xi1 guan3, “sucker”) | 雪人 (xue3 ren2, “snowman”) | 钟表 (zhong1 biao3, “clock”) | 雪人 (xue3 ren2, “snowman”) |
