# Supplementary material for: The Role of Phoneme in Mandarin Chinese Production: Evidence from ERPs
Source: PLoS One. 2014 Sep 5;9(9):e106486. doi: 10.1371/journal.pone.0106486 (PMC4156350; doi:10.1371/journal.pone.0106486)
Supplement: Appendix S2 — Stimuli used in Experiment 2. (DOC) [file pone.0106486.s002.doc]

**Appendix S2: Stimuli used in Experiment 2.** Numbers represent tones and neutral tone is not marked.

| Phonologically related prime-target pairs | | Phonologically unrelated prime-target pairs | |
| --- | --- | --- | --- |
| 教堂 (jiao4 tang2, “church”) | 抽屉(chou1 ti4, “drawer”) | 背心 (bei4 xin1, “waistcoat”) | 抽屉(chou1 ti4, “drawer) |
| 风车 (feng1 che1, “windmill”) | 小丑 (xiao3 chou3, “clown”) | 戒指 (jie4 zhi, “ring”) | 小丑 (xiao3 chou3, “clown”) |
| 烟囱 (yan1 cong1, “chimney”) | 蔬菜 (shu1 cai4, “vegetable”) | 熨斗 (yun4 dou3, “iron”) | 蔬菜 (shu1 cai4, “vegetable”) |
| 车轮 (che1 lun2, “wheel”) | 铁链 (tie3 lian4, “chain”) | 信封 (xin4 feng1, “envelop”) | 铁链 (tie3 lian4, “chain”) |
| 飞机 (fei1 ji1, “airplane”) | 书架 (shu1 jia4, “bookshelf”) | 蛋糕 (dan4 gao1, “cake”) | 书架 (shu1 jia4, “bookshelf”) |
| 手枪 (shou3 qiang1, “gun”) | 地球 (di4 qiu2, “globe”) | 水壶 (shui3 hu2, “kettle”) | 地球 (di4 qiu2, “globe”) |
| 衬衫 (chen4 shan1, “shirt”) | 袋鼠 (dai4 shu3, “kangaroo”) | 草莓 (cao3 mei2, “strawberry”) | 袋鼠 (dai4 shu3, “kangaroo”) |
| 熨斗 (yun4 dou3, “iron”) | 领带 (ling3 dai4, “tie”) | 教堂 (jiao4 tang2, “church”) | 领带 (ling3 dai4, “tie”) |
| 酒杯 (jiu3 bei1, “wineglass”) | 毛笔 (mao2 bi3, “paintbrush”) | 风车 (feng1 che1, “windmill”) | 毛笔 (mao2 bi3, “paintbrush”) |
| 蛋糕 (dan4 gao1, “cake”) | 吸管 (xi1 guan3, “sucker”) | 烟囱 (yan1 cong1, “chimney”) | 吸管 (xi1 guan3, “sucker”) |
| 草莓 (cao3 mei2, “strawberry”) | 斑马 (ban1 ma3, “zebra”) | 车轮 (che1 lun2, “wheel”) | 斑马 (ban1 ma3, “zebra”) |
| 背心 (bei4 xin1, “waistcoat”) | 螃蟹 (pang2 xie4, “crab”) | 飞机 (fei1 ji1, “airplane”) | 螃蟹 (pang2 xie4, “crab”) |
| 戒指 (jie4 zhi, “ring”) | 蜡烛 (la4 zhu2, “candle”) | 手枪 (shou3 qiang1, “gun”) | 蜡烛 (la4 zhu2, “candle”) |
| 头盔 (tou2 kui1, “helmet”) | 扑克 (pu1 ke4, “cards”) | 衬衫 (chen4 shan1, “shirt”) | 扑克 (pu1 ke4, “cards”) |
| 信封 (xin4 feng1, “envelop”) | 头发 (tou2 fa4, “hair”) | 蜗牛 (wo1 niu2, “snail”) | 头发 (tou2 fa4, “hair”) |
| 蜗牛 (wo1 niu2, “snail”) | 电脑 (dian4 nao3, “computer”) | 酒杯 (jiu3 bei1, “wineglass”) | 电脑 (dian4 nao3, “computer”) |
| 水壶 (shui3 hu2, “kettle”) | 小号 (xiao3 hao4, “trumpet”) | 浴盆 (yu4 pen2, “bathtub”) | 小号 (xiao3 hao4, “trumpet”) |
| 浴盆 (yu4 pen2, “bathtub”) | 大炮 (da4 pao4, “cannon”) | 头盔 (tou2 kui1, “helmet”) | 大炮 (da4 pao4, “cannon”) |
